# Remote whispering metamaterial for non-radiative transceiving of ultra-weak sound

**DOI:** 10.1038/s41467-021-23991-3

**Published:** 2021-06-16

**Authors:** Jin Zhang, Wei Rui, Chengrong Ma, Ying Cheng, Xiaojun Liu, Johan Christensen

**Affiliations:** 1 Key Laboratory of Modern Acoustics, Department of Physics and Collaborative Innovation Center of Advanced Microstructures, Nanjing University, Nanjing, China; 2 Department of Physics, Universidad Carlos III de Madrid, Leganés, Madrid Spain

**Keywords:** Metamaterials, Acoustics

## Abstract

Transceiving ultra-weak sound typically relies on signal pre-amplification at the transmitting end via active electro-acoustic devices, which inherently perturbs the environment in the form of noise that inevitably leads to information leakage. Here we demonstrate a passive remote-whispering metamaterial (RWM) enabling weak airborne sound at audible frequencies to reach unprecedented signal enhancement without altering the detected ambient soundscape, which is based on the extraordinary scattering properties of a metamaterial formed by a pair of self-resonating subwavelength Mie meta-cavities, constituting the acoustic analogy of Förster resonance energy transfer. We demonstrate efficient non-radiative sound transfer over distances hundreds times longer than the radius of the meta-cavities, which enables the RWM to recover weak sound signals completely overwhelmed by strong noise with enhanced signal-to-noise ratio from −3 dB below the detection limit of 0 dB in free space to 17.7 dB.

## Abstract (web summary)

Typically, sending sound from transmitter to receiver requires pre-amplification and disturbs the surrounding sound environment. Here, the authors present a metamaterial designed to enable transmission of weak sound that can be recovered even in the presence of strong noise

## Introduction

**Fig. 1:**
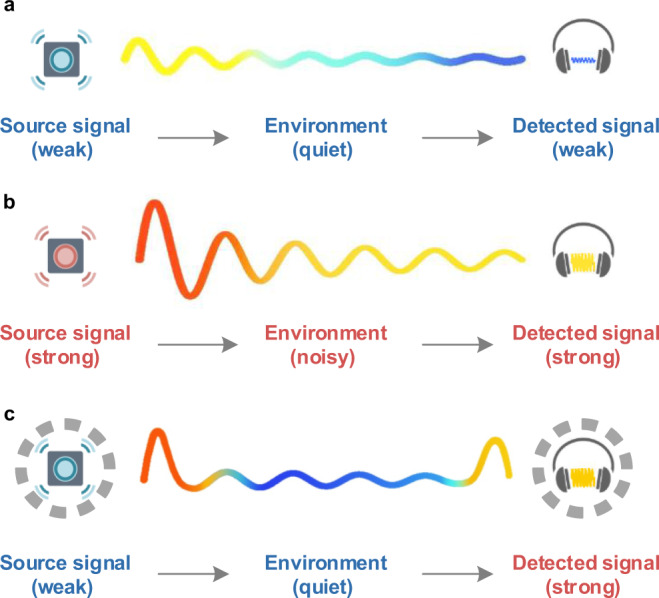
Conventional and enhanced sound transception. **a** Conventional scheme transceiving weak sound. The detected signal at the receiving end is much weaker after distant spread while the ambient environment remains quiet. **b** Conventional scheme transceiving strong sound. The detected signal is enhanced but meanwhile the ambient environment turns noisy. **c** Scheme based on the use of the remote whispering metamaterial. Enhanced emission and reception of a weak sound signal, at which the pressure field of sound is enhanced only near the emitting and receiving sites. Note that the detected signal is strong while ambient soundscape remains quiet.

Transferring weak sound efficiently is of particular importance in various acoustic technologies, including sonar systems^1^, speech communications^2^, medical imaging^3^, acoustic surveillance^4^, and many more. In classical acoustic theory, the amplitude diminishes in a free-field environment due to geometric spreading and losses, which critically limit the detection capability of sound transceivers. For the often-used conventional sound transfer scheme shown in Fig. 1a, b, the intensity of detected sound at the receiving end is proportional to the one of the source at the emitting end. As a consequence, one must ensure a strong enough source [see Fig. 1b] in order to maintain detectable signal intensities with sufficient signal-to-noise ratio (SNR) at remote locations, especially in noisy backgrounds. In order to avoid overhearing of secret information or preventing to disturb other listeners, people whisper quietly to accomplish this. However, privy listeners face no advantage in the presence of a noisy soundscape. Hence, when the sound source is weak relative to, e.g., surrounding speech noise, pre-amplification of remote acoustic signals via active electro-acoustic devices (i.e., power amplifier and loudspeaker) is necessary^5,6^.

Over the past decade, significant research efforts have been devoted to exploring acoustic metamaterials for efficient sound transfer enhancement. Acoustic metamaterials are artificial man-made materials enabling unusual wave properties and responses not found in nature^7^. Some pioneering metamaterials-based devices have been demonstrated to manipulate the processes of sound emission^8–14^, propagation^15–26^ or reception^27,28^, respectively, such as emission power enhancement of a speaker inside a Mie meta-cavity via the acoustic Purcell effect^8^, directional sound radiation by phononic crystals or locally resonant metamaterials to achieve beamforming^9,10^, and signal amplification by anisotropic tapered metamaterials via a wave compression effect^27^. However, most previous works focused on a single operation instead of looking at the transceiving processes, let alone the radiative interaction between the sound waves and the surrounding environment. Recently, non-radiative wireless electromagnetic power transfer using magnetic resonance coupling has been explored^29–34^, enabling promising routes toward novel applications for wireless powering of implantable medical devices and electric vehicles. In traditional acoustics, although two near-field resonant objects tuned at the same resonance frequency also tend to exchange energy efficiently^15^, the fundamental question remains: does an acoustic analog of non-radiative wireless power transfer exist?

In this work, we explore the use of acoustic metamaterials as a means to realize distant and silent transfer of weak sound to targeted individuals or locations. The idea is to maintain the concept (privacy) of whispering, but to do this remotely from an ultra-weak sound source not located near the targeted receiver. Here, we propose a remote whispering metamaterial (RWM) scheme that incorporates a pair of coupled Mie resonant objects around both the weak source and the receiver site at a deep-subwavelength scale [see Fig. 1c]. Such system allows spatial concentration of wave energy and induces strong pressure enhancement only inside the object pair, which enables multiplying enhanced emission and reception of weak sound signals while maintaining a quiet environment. As a proof of concept, we predict theoretically and demonstrate experimentally the efficiency of the RWM using artificial Mie resonant meta-cavities constructed out of a subwavelength maze-like structure. By using such a metamaterial device, non-radiative sound transfers over distances up to 32.5 times the radius of the meta-cavities is successfully demonstrated with more than 40 dB enhancement of the detected signal and an average −20 dB reduction of the ambient sound leakage compared to ordinary setups.

## Results

### Theoretical model

**Fig. 2:**
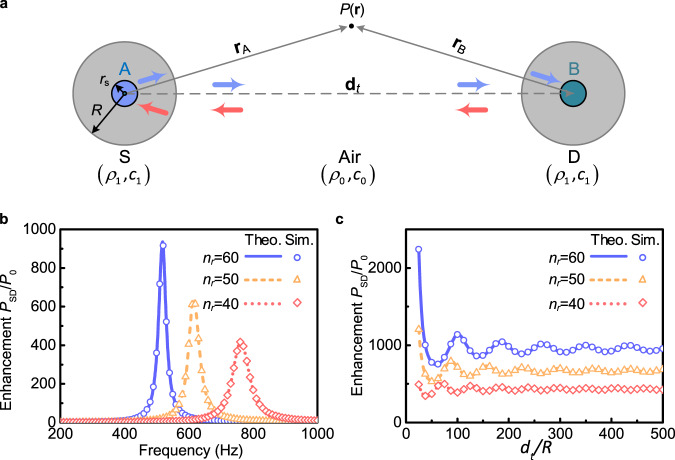
Theoretical model of the RWM system. **a** Schematic of the system setup. A is a single point-like sound source of radius *r*_s_ emitting a sine harmonic wave. B is a condensed microphone, which picks up the sensing signal. *S* and *D* are the high-refractive-index (*n*_r_ = *c*_0_/*c*_1_ > 1) elements enclosing the source and detector, respectively. **b** Theoretical and simulated transfer enhancement from A to B using the RWM. Here the transfer distance *d*_t_ is 325 times the element radius *R*. **c** Enhancement at different distances *d*_t_.

We first focus on a simplified configuration of two objects for point-to-point sound transfer, as shown in Fig. 2a. One emitting object (identified by the subscript S) is driven internally at a constant frequency by the enclosed ultra-weak monopole sound source (subscript A). Sound is detected by the other receiving object (subscript D) by means of an ordinary condensed microphone (subscript B), which picks up the signals. Both cylindrical objects of subwavelength radius *R* that possess identical effective high-refractive indices *n*_r_ relative to the background medium air (to guarantee strong field confinement) are separated by a large distance *d*_t_ (*d*_t_ ≫ *R*). The density and sound speed of background medium air are *ρ*_0_ and *c*_0_, while those of cylindrical objects are *ρ*_1_ and *c*_1_, respectively. Here *c*_1_ is remarkably lower than *c*_0_. Intuitively, the physical system representing sound transfer between resonant objects strongly coupled to one another can be described using the coupled-mode theory, which has been extensively studied in short-range and mid-range wireless electromagnetic power transfer systems^35–38^. In order to demonstrate the effect of the RWM universally, we develop a theoretical model on the basis of a rigorous acoustic scattering theory, which applies well for both the near-field and far-field configurations. Ensuring the continuity of both the pressure *P*(*r*) and particle velocity 1/*ρ* · ∂*P*/∂*r* at the interfaces, the ehancement of the measured pressure value by the high-refractive-index elements from the point A to B is given by (see Supplementary Note 1 for details):

1 $$\eta =\frac{{\rho }_{1}|{{\bf{T}}}_{G\alpha }|}{{\rho }_{0}|{\bf{T}}||{H}_{0}({k}_{0}{d}_{t})|}$$

where *k*_0_ denote the wavenumbers in the air, *H*_*m*_ is the Hankel functions of the first kind. **T** and **T**_*Gα*_ are relation matrices obtained from equation (3) in Supplementary Note 1. |**T**| refers to the determinant of a matrix.

Figure 2b depicts the spectra of the enhancement efficiency *P*_SD_/*P*_0_ for various refractive indices *n*_r_, with *c*_0_ = *n*_r_*c*_1_, *R* = 4 mm and *d*_*t*_ = 1.3 m. Full-wave simulations by finite element method were also performed using the pressure-acoustic and thermo-acoustic module in COMSOL Multiphysics software. The simulated results (symbols) are found to be in very good agreement with the theoretical predictions (curves). For the cases of *n*_r_ = 40, 50, and 60, the spectra exhibit maximum enhancement peaks of 418, 646, and 945 at the frequencies of 761, 614, and 518 Hz, respectively, corresponding to substantial detected amplified signals of 52, 56, and 59 dB, respectively. It is worth noting that the maximum enhancement increases with the increment of *n*_r_ while the peak of interest shifts to lower frequencies correspondingly. A consequence of this is that a compact element with higher *n*_r_ value can acquire a significantly improved sound signal at a target position. In order to further confirm the system performance at different propagation distances, Fig. 2c shows the enhancement efficiency with increasing distance *d*_t_, ranging from the near to the far field. The enhancement efficiency oscillates at close range but become steady as *d*_t_ increases. We also demonstrate the efficiency of the RWM at higher-order monopolar modes (Supplementary Note 2). Note that the strong monopole–monopole resonance interaction between the emitter/receiver pair plays an important role in the near-field when both are placed in close proximity, and the emitter’s acoustic spectrum overlaps exactly with the receiver’s. Such factors are reminiscent to the short-range dipole–dipole interaction between fluorescent emitters, i.e., Förster Resonant Energy Transfer^39^, whose application is important for detecting molecular dynamics in biophysics and biochemistry. In theory, the enhancement grows with increase of the refractive index *n*_r_ [see Fig. 2b, c], however, inherent thermo-viscous losses at higher indices in RWM made of even finer coiled-up micro-structure should hinder the potentially achievable amplification levels. Consequently, there is a trade-off between enhancement levels and designing high index RWMs.

### Demonstration of remote whispering

**Fig. 3:**
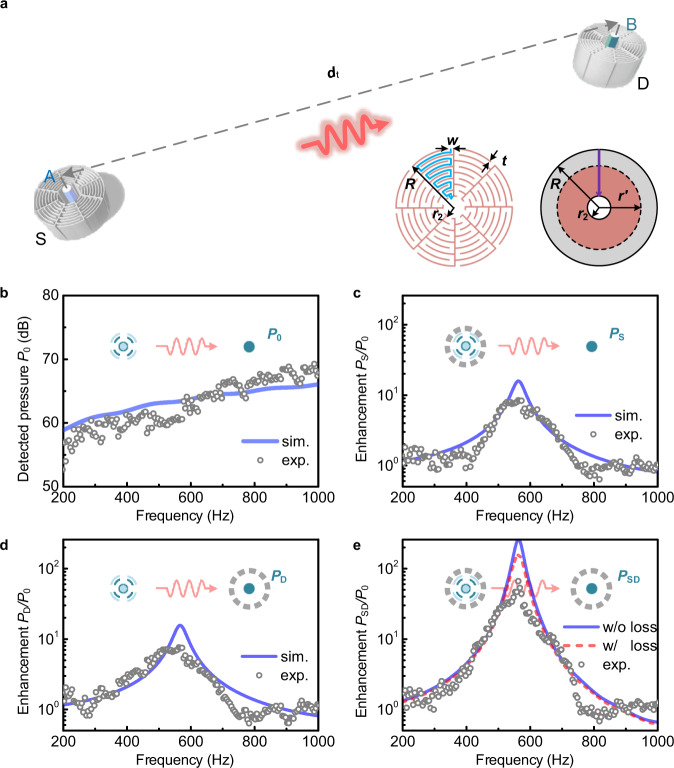
Physical demonstration of the RWM system. **a** Schematic illustration. *S* and *D* refer to the source and detector meta-cavities, respectively. The amplitude of source A is adjusted to ensure that the direct radiative transfer from A to B is negligible. Inset: schematic cross-sectional view of the meta-cavity (left) and its equivalent physical model (right). **b** Detected pressure *P*_0_ for direct transfer from A to B without meta-cavities *S* and *D* [refer to Fig. 1a, b]. Detected pressure enhancement from A to B for **c** *P*_*S*_/*P*_0_ with S only, **d** *P*_*D*_/*P*_0_ with D only, and **e** *P*_SD_/*P*_0_ with both *S* and *D* [refer to Fig. 1c]. Here the transfer distance *d*_*t*_ is 32.5 times the radius of the meta-cavities.

Our physical realization of the RWM consists of two subwavelength meta-cavities [Fig. 3a] sustaining intense Mie self-resonances for applicable for transceiving enhancement, which are fabricated with epoxy resin by means of 3D printing^17^. The left inset shows the cross-sectional view of a meta-cavity with outer (inner) radius *R* (*r*_2_), which is uniformly divided into eight sections each made of an eightfold zig-zag channel aiming to multiply the equivalent sound path therein. The width and thickness of the channels are *w* = 0.08*R* and *t* = 0.02*R*, respectively. This labyrinthine configuration possesses an extraordinary high-refractive-index *n*_r_ = 4.55 relative to the background medium air as the acoustic waves are forced to travel along the extended zig-zag channels, which can be approximately calculated as the length ratio of the blue zig-zag path and the purple straight line.

In order to demonstrate the practical use of the RWM, we begin investigating two separate cases and one coupled RWM pair. Figure 3c, d show the detected pressure enhancement *P*_S_/*P*_0_ and *P*_D_/*P*_0_ transferred from A to B in the presence of either meta-cavity *S* or *D* only, respectively, measured at the receiving location, 1.3 m (=32.5*R*) away from point-like source. *P*_0_ is the detected pressure for direct transfer from A to B in free space [Fig. 3b]. The pressure amplification that reaches over an order in magnitude (≈15.8, 24 dB) has been achieved in both scenarios at the frequency of *f*_0_ = 563 Hz, as the well-agreeing measurement data and simulations confirm. Beyond that, the radiation resistance of the source is significantly enhanced in the presence of the RWM, see Supplementary Note 3. For comparison, the pressure enhancement spectra are plotted in Fig. 3e when the RWM is incorporated with both the source and the detector. As the predicted results indicate, the strongly coupled meta-cavities exhibit highly efficient non-radiative sound transfer with pressure amplification at over two orders in magnitude (≈259, that is, 48 dB). The deviation from the measured peak, i.e., a 66-fold enhancement, should be ascribed to the viscous and thermal power dissipation, which gives rise to a factor of 157 for lossy computations shown by the red-dashed curve in Fig. 3e. For the experimental setup and measurement, we employed a balanced armature speaker (Knowles, Model ID DWFK-31785-000, 5 × 2.7 × 3.9 mm^3^) as the monopole source (see Supplementary Note 4 for details). Lastly, our theoretical approximation using a rigorous acoustic scattering theory has led to closed form analytical expressions to characterize the spatial and spectral pressure field distributions (Supplementary Note 5). The theoretical results clearly confirm the numerical simulations and experimental measurements. In the near-field regime, the enhancement when both elements are enclosed by the RWM exceeds the product of the individual processes since *P*_SD_*P*_0_ > *P*_S_*P*_D_, which stems from the strong monopole–monopole interaction occurring between two such resonators that are giving rise to the pronounced sound field enhancement including pressure oscillations. On the other hand, in the far-field the system can be broken down into two cascaded processes of emission and receiving the signal, and the total transfer efficiency is approximately the product of two efficiencies, *P*_SD_*P*_0_ ≈ *P*_S_*P*_D_ (Supplementary Note 5). Moreover, the proposed Mie meta-cavities exhibit a superior robustness compared to classic Helmholtz resonators. (Supplementary Note 6).

### Whispering weak sound to remote targets

**Fig. 4:**
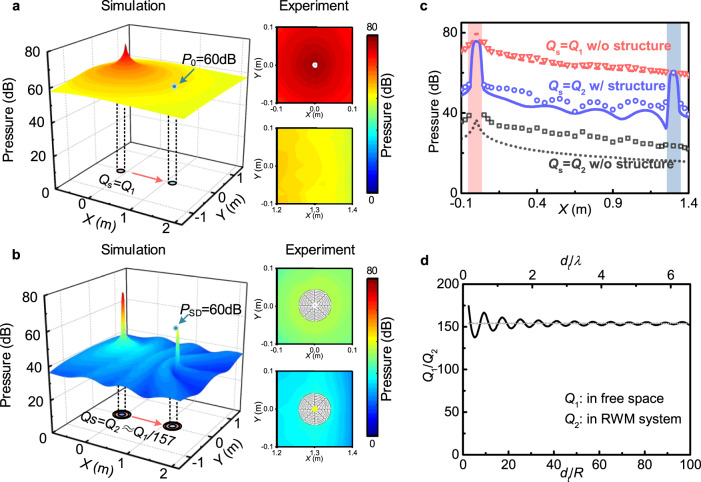
Whispering weak sound to remote targets. Sound pressure fields of (**a**) a strong sound source of intensity *Q*_1_ in free space, and (**b**) a weak source of intensity *Q*_2_ = *Q*_1_/157 using the proposed system. The detected pressure at the receiving points reaches 60 dB in both cases. The insets in **a**, **b** show experimental pressure fields around the emitting and receiver sites. **c** Corresponding profiles of sound pressure along *x*(*m*) for *y* = 0 in three cases: the strong source *Q*_1_ in free space (red), the weak source *Q*_2_ with the RWM (blue), and a weak source *Q*_2_ in free space for comparison (gray). The curves and symbols denote calculated and measured results, respectively. **d** The source intensity contrast of *Q*_1_/*Q*_2_ at different distances to maintain a 60 dB pressure level detected at the receiving point with the RWM and in free space.

The remote whispering process allows one to focus highly amplified acoustic signals originating from a weak sound source to specific remote locations while leaving the surrounding soundscape unaltered thanks to the non-radiative characteristics. For illustration, as shown in Fig. 4a, a strong point-like sound source with a volume flow rate per unit length *Q*_S_ = *Q*_1_ = 0.79 mm^2^ s^−1^ is placed at the emitting end such that the detected sound pressure at the receiving location at a distance of *d*_t_ = 32.5*R* away from the source is *P*_0_ = 60 dB. Here the intensity *Q*_S_ of the source was adjusted to obtain *P*_0_ at 60 dB, which is about the level of ordinary indoor conversations. It is seen that the sound pressure of the ambient environment is always higher than at the receiving location, which means the signal can also be detected elsewhere and the whole space becomes noisy. In comparison, Fig. 4b shows the unique performance when utilizing the RWM system. Here, we employed a much weaker sound source of reduced intensity *Q*_2_ = *Q*_1_/157 while the listener still is set to detect incoming sound pressure at 60 dB level. Interestingly, the environmental interference is dramatically reduced thanks to very low-pressure levels throughout the entire space surrounding the peaking sites of the sound emitter and receiver.

We further quantify the radiationless sound transfer by plotting corresponding sound pressure profiles along a straight path from the source to the receiver as shown in Fig. 4c. The red, blue, and gray curves (symbols) represent the calculated (experimental) results of the cases with a strong source *Q*_1_ in free space [Fig. 4a], a weak source *Q*_2_ using the RWM configuration [Fig. 4b], and a weak source *Q*_2_ in free space (see Supplementary Note 7 for details), respectively. The RWM configuration clearly exhibits more than 40 dB enhancement of the detected signals and an average of −20 dB in reduction of the ambient sound leakage compared to the ordinary setups. Figure 4d shows the source intensity contrast of *Q*_1_/*Q*_2_ at different distances to maintain an identical pressure level detected at the receiving location with and without the RWM configuration. When the meta-cavities are in close proximity the intensity contrast display oscillation due to the strong near-field, however when pulled apart, the intensity ratio becomes steady at 157. Based on the deeply subwavelength nature of the RWM, i.e., *R* = 0.04 m, compared to the operating wavelength of *λ* = 0.61 m, it can readily be idealized as a so-called “point source” with a clear distinction between near- and far-field. Specifically, the near-field constitutes the region within a radius *d*_*t*_ ≪ *λ*, while the far-field emerges at *d*_*t*_ ≫ 2*λ*. Thus, we conclude that the coupling and the enhancement remain strong in both regions. The enhancement efficiency oscillates with large amplitudes when *d*_t_ is small since the strong short-range monopole–monopole resonance interaction between emitter/receiver meta-cavities plays an important role when they are placed in close proximity where evanescent waves amplify. In this context, this near-field behavior is understood through a non-radiative mechanism. Contrary, in the far-field, when the meta-cavities are pulled apart, the short-range evanescent-wave coupling between the two resonators can be neglected and the enhancement efficiency becomes steady. Thus, here one is capable to transfer sound over extended distances without altering the detected ambient soundscape in a non-radiative manner, since the radiated field concentrates only inside the receiver while it remains at low levels in the surrounding area, which is obviously different from that of an ordinary radiative system, especially in the far-field [see Fig. 4a, b]. In addition, we would like to emphasize that the transfer efficiency is robust against variations of operating conditions, e.g., introducing either large-sized solid obstacles or scattering layers consisting of various randomly distributed rigid scatterers in the propagation path between the emitting and receiving ends (Supplementary Note 8). We also demonstrate multi targeted remote whispering in which weak sound signals are transferred to different locations in a quiet and efficient fashion (Supplementary Note 9). Moreover, the enhancement efficiency *P*_SD_/*P*_0_ characterizes the magnification of the measured sound pressure with and without the RWM. Naturally, the sound pressure decays with distance as 1/*d*_t_, thus from a practical point of view, the measured amplified pressure decreases with growing distance *d*_t_. Hence, a compromise between a steady enhancement and the available acoustic energy at a distant point must be reached.

### Anti-interference remote-whispering in a noisy environment

**Fig. 5:**
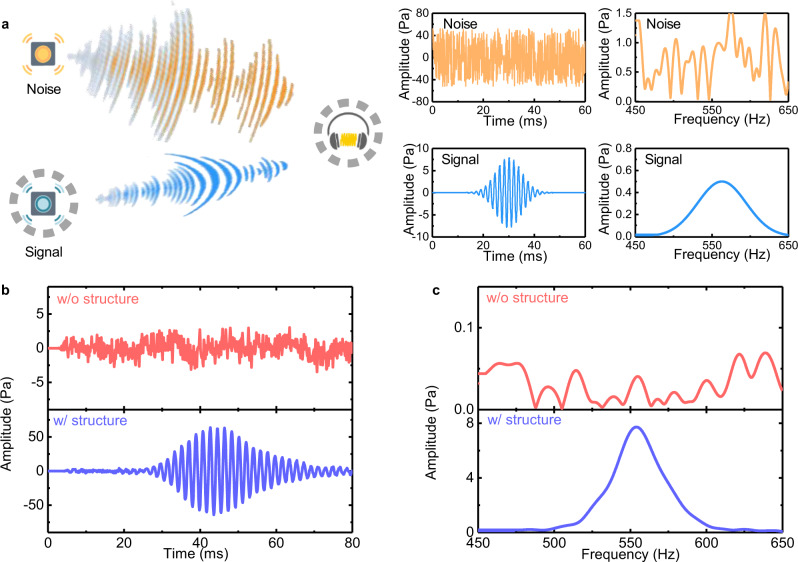
Anti-interference remote whispering in a noisy environment. **a** Schematic setup: a weak sound source emits a series of Gaussian modulated acoustic pulses with center frequency at 563 Hz and bandwidth of 75 Hz (blue). The strong external interference source radiates broadband white noise (yellow) to mask the desired weak signal. Insets: pressure waveforms in the time domain and corresponding FFT spectra of the noise and signal, respectively. **b** Pressure waveforms in the time-domain and **c** corresponding FFT spectra obtained in free space without the RWM (upper panel) and with the RWM (lower panel) structure.

We demonstrate that in addition to the reduced impact on the environment, the proposed RWM system can also capture weak acoustic signals that are overwhelmed by a spatially separated intensive noise sources. As shown in Fig. 5a, a series of Gaussian modulated sinusoidal pulses with a center frequency at 563 Hz and a bandwidth of 75 Hz is generated from the speaker (blue), which mimics a whispered sound signal. We introduce a strong external interference source radiating broadband white noise (yellow) to mask the whispered weak signal, and investigate how the RWM can eliminate this interference through self-selecting and amplifying the source signal at the operating frequency. The time-domain waveforms and corresponding fast-Fourier-transform (FFT) spectra of the input noise and whispered signal are shown in the insets of Fig. 5a. Note that the level of noise is about five times larger in amplitude compared to the emitted Gaussian signal, which is much below the detection limit of conventional acoustic sensing system (SNR < 1). Figure 5b, c shows the time domain and frequency domain pulse signal in free space (top) and by using the RWM (bottom). The results clearly demonstrate that the detected signal in free space is completely overwhelmed by dominating noise with a SNR approaching only 0.5, which prevents the signal to be effectively detected. On the contrary, the same weak signal can be restored efficiently when employing the RWM in which the signal is amplified more than 100 times while the waveform does not suffer from any obvious distortions. Hence, thanks to the RWM, the acoustic information perceived by the recipient can be captured from the weak source in the absence of undesired strong noise. Here, the SNR has an increment of more than 20 dB (from 0.5 to 58.4, i.e., more than 2 order in magnitude enhancement above the detection limit), which is determined by the total power *W* (*W*_0_, the noise floor) within the range 525.5–600.5 Hz when the source speaker is turned on (off), that is, SNR = (*W* – *W*_0_)/*W*_0_. The enhancement of the SNR is consistent under different background noise conditions (Supplementary Note 10), and the RWM system shows good performance even when applied in an extreme scenario in which a highly noisy source is placed in between a nearby listener and a distant weak source signal (Supplementary Note 11). We have also evaluated the SNR in dependence to the receiver area. The results indicate a superior detection of weak sound signals within the limiting bounds of conventional acoustic detection systems, confirming the remarkable high transfer efficiency of the RWM for sound signal and information (Supplementary Note 12). These results demonstrate the capability to conduct anti-interference remote whispering for weak signals with metamaterials in a highly noisy environment. In addition, certain limited reconfigurations of the device can be provided even if the physical structure of the RWM remains unchanged, which is highly desirable for practical implementations (Supplementary Note 13).

## Discussion

In conclusion, we proposed theoretically and demonstrated experimentally a RWM scheme made of coupled Mie resonators capable to enhance non-radiative sound transfer over extended distances. Our RWM system emits sound quietly to remote locations in the far-field, which relinquishes the need for strong sources while still maintaining high transfer efficiency. The detected sound pressure using our RWM is enhanced by more than 100 times (that is, >40 dB) compared to free space. Thus, the RWM system shows unique performance and functionalities, including preserved quiet soundscapes and stronger anti-jamming capability compared to current acoustic transmission devices. Although we mainly focused on the case using a speaker in a two-dimensional setup comprising the cylindrical structure, we foresee that the use of an effective high-refractive-index RWM may enable new technological avenues with diverse geometrical configurations. For example, previously three-dimensional spherical and cubic coiled-elements were reported in the literature^40,41^. Although these reports targeted different use, extending our RWM approach by using three-dimensional Mie resonators could potentially enable enhanced communications when enclosing someone speaking in a microphone. Moreover, although we mainly focused on an airborne sound implementation, the concept can also enable flexible realization of signal remote whispering transfer in more general systems such as underwater environment (Supplementary Note 14). The RWM could also be considered for microwaves working under the same principle (Supplementary Note 15). Hence, we expect that the proposed RWM device will offer rich opportunities to advance metamaterial technologies for novel functionalities.

## Supplementary information

Supplementary Information

## Data Availability

The data that support the findings of this study are available from the corresponding author upon reasonable request.

## Code availability

The code used to calculate the results for this work is available from the corresponding author upon reasonable request.
