# Supplementary material for: Remote whispering metamaterial for non-radiative transceiving of ultra-weak sound
Source: Nat Commun. 2021 Jun 16;12:3670. doi: 10.1038/s41467-021-23991-3 (PMC8208968; doi:10.1038/s41467-021-23991-3)
Supplement: Supplementary file 1 — Supplementary Information [file 41467_2021_23991_MOESM1_ESM.pdf]

Supplementary Material for the paper

Remote Whispering Metamaterial for Non-radiative Transceiving of Ultra-weak Sound

Jin Zhang[1], Wei Rui[1], Chengrong Ma[1], Ying Cheng[1,*], Xiaojun Liu[1,†] & Johan Christensen[2,‡]

[1] *Key Laboratory of Modern Acoustics, Department of Physics and Collaborative Innovation Center of Advanced Microstructures, Nanjing University, Nanjing 210093, China*

[2] *Department of Physics, Universidad Carlos III de Madrid, 28916 Leganés, Madrid, Spain.*

Email: * chengying@nju.edu.cn; † liuxiaojun@nju.edu.cn; ‡ johan.christensen@uc3m.es

Note 1. Theoretical model on the basis of a rigorous acoustic scattering theory 2

Note 2. RWM at higher-order monopolar modes 4

Note 3. Influence on radiation impedance of the speaker 5

Note 4. Experimental setup 6

Note 5. Describing the physical model with a rigorous acoustic scattering theory 7

Note 6. Comparison with the classic acoustic Helmholtz resonators 10

Note 7. Sound pressure field emitted from a weak sound source in free space 12

Note 8. System robustness 13

Note 9. Multi-target remote whispering 15

Note 10. Overcoming the conventional acoustic detection limit 16

Note 11. Anti-interference remote whispering in an extreme case 17

Note 12. High transfer efficiency for sound signal & information 18

Note 13. System reconfigurability 21

Note 14. RWM system for underwater sound 22

Note 15. RWM system for electromagnetic signal 23

Supplementary References 24

Note 1. Theoretical model on the basis of a rigorous acoustic scattering theory

Setting $\mathbf{r}=(x,y)=(r,\theta)$, the pressure field $P(\mathbf{r})$ obeys the 2D Helmholtz equation $(\nabla^2+k^2)P=0$, which can be decomposed into the incident and scattering cylindrical wave components represented by Bessel (J_m) and Hankel (H_m) functions： $P(\mathbf{r})=\sum_m A_m H_m(k_1|\mathbf{r}_\mathrm{A}|)+B_m J_m(k_1|\mathbf{r}_\mathrm{A}|)$ in the interior of element S (the region $r_s<|\mathbf{r}_\mathrm{A}|<R$); $P(\mathbf{r})=\sum_m C_m H_m(k_0|\mathbf{r}_\mathrm{A}|)+D_m J_m(k_0|\mathbf{r}_\mathrm{A}|)=\sum_m E_m J_m(k_0|\mathbf{r}_\mathrm{B}|)+F_m H_m(k_0|\mathbf{r}_\mathrm{B}|)$ in the exterior of both elements (the region $|\mathbf{r}_\mathrm{A}|>R\ \cap\ |\mathbf{r}_\mathrm{B}|>R$); $P(\mathbf{r})=\sum_m G_m J_m(k_1|\mathbf{r}_\mathrm{B}|)$ in the interior of element D (the region $r_\mathrm{B}<R$). Here $\mathbf{r}_\mathrm{A}$ and $\mathbf{r}_\mathrm{B}$ are position vectors of the same point in corresponding local coordinate systems, with point A and point B as the coordinate origins, respectively. A_m~G_m are the mth-order expansion coefficients to be determined; k_0 and k_1 denote the wavenumbers in the air and the high-refractive-index elements, respectively. In the exterior space, the incident waves of the element D originate from the outgoing waves of the element S and vice versa, which can be expressed as $\sum_m E_m J_m(k_0|\mathbf{r}_\mathrm{B}|)=\sum_m C_m H_m(k_0|\mathbf{r}_\mathrm{A}|)$ and $\sum_m D_m J_m(k_0|\mathbf{r}_\mathrm{A}|)=\sum_m F_m H_m(k_0|\mathbf{r}_\mathrm{B}|)$. Such reciprocal relationship can be characterized by coordinate transformations:

$$\begin{cases}\sum_m C_m H_m(k_0|\mathbf{r}_\mathrm{A}|)=\sum_m\left[\sum_n C_n H_{m-n}(k_0|\mathbf{d}_t|)e^{jm\emptyset_\mathrm{B}+j(m-n)\emptyset_d}\right]J_m(k_0|\mathbf{r}_\mathrm{B}|)\\ \sum_m F_m H_m(k_0|\mathbf{r}_\mathrm{B}|)=\sum_m\left[\sum_n F_n H_{m-n}(k_0|\mathbf{d}_t|)e^{jm\emptyset_\mathrm{A}-j(m-n)\emptyset_d}\right]J_m(k_0|\mathbf{r}_\mathrm{A}|)\end{cases}, \quad (1)$$

where $\emptyset_\mathrm{A}$, $\emptyset_\mathrm{B}$ and $\emptyset_{d_t}$ are phase angles of vectors $\mathbf{r}_\mathrm{A}$, $\mathbf{r}_\mathrm{B}$ and $\mathbf{d}_t$, respectively. $|\mathbf{d}_t|$ refers to the norm of the vector $\mathbf{d}_t$. Considering the case of monopole resonance, the 0th-order coefficient far exceeds the higher-order coefficients, thus Eq. (1) can be approximated as:

$$\begin{cases}E_0\approx C_0H_0(k_0|\mathbf{d}_t|)\\ D_0\approx F_0H_0(k_0|\mathbf{d}_t|)\end{cases}, \quad (2)$$

Combining the continuities of $P(r)$ and $1/\rho\cdot\partial P/\partial r$ at the interfaces $|\mathbf{r}_\mathrm{A}|=R$ and $|\mathbf{r}_\mathrm{B}|=R$, the 0th-order coefficients are obtained by solving:

$$\mathbf{T}\times[B_0\quad C_0\quad D_0\quad E_0\quad F_0\quad G_0]^T=A_0\boldsymbol{\alpha} \quad (3)$$

where $\boldsymbol{\alpha}=\left[H_0(k_1R)\quad \frac{k_1}{\rho_1}H_0'(k_1R)\quad 0\quad 0\quad 0\quad 0\right]^T$ and the matrix $\mathbf{T}$ is given by:

$$\mathrm{T}=\begin{bmatrix}-J_0(k_1R) & H_0(k_0R) & J_0(k_0R) & 0 & 0 & 0\\ -\frac{k_1}{\rho_1}J_0'(k_1R) & \frac{k_0}{\rho_0}H_0'(k_0R) & \frac{k_0}{\rho_0}J_0'(k_0R) & 0 & 0 & 0\\ 0 & 0 & 0 & J_0(k_0R) & H_0(k_0R) & -J_0(k_1R)\\ 0 & 0 & 0 & \frac{k_0}{\rho_0}J_0'(k_0R) & \frac{k_0}{\rho_0}H_0'(k_0R) & -\frac{k_1}{\rho_1}J_0'(k_1R)\\ 0 & H_0(k_0d_t) & 0 & -1 & 0 & 0\\ 0 & 0 & -1 & 0 & H_0(k_0d_t) & 0\end{bmatrix}. \quad (4)$$

Note that owing to the monopolar resonance nature, the pressure value at the receiving position $|\mathbf{r}_\mathrm{B}|=0$ should be $P_\mathrm{SD}=\sum_m G_m J_m(0)=G_0$, which equals to the 0th-order

expansion coefficient of the incident waves in element D. As a consequence, we obtain the following detected pressure as:

$$P_{\text{SD}} = G_0 = \frac{|\mathbf{T}_{G\alpha}|}{|\mathbf{T}|} A_0 \tag{5}$$

where $\mathbf{T}_{G\alpha}$ is the matrix obtained by replacing the column in $\mathbf{T}$ corresponding to G_0 by the elements in vector $\boldsymbol{\alpha}$. $|\mathbf{T}|$ refers to the determinant of a matrix. A_0 is the coefficient determined by the monopole source located in the element S. On the other hand, the detected pressure for sound radiation from identical source in the free space is $P_0 = A_0' H_0(k_0|\mathbf{d}_t|)$. Considering a monopolar point source ($r_s \to 0$) whose boundary condition is $v_r|_{r=r_s} = u_a e^{j\omega t}$, the coefficicient A_0 and A_0' becomes $A_0 = -\frac{k_1 H_0'(k_1 r_s)}{j\omega\rho_0} u_a e^{j\omega t}$ and $A_0' = -\frac{k_0 H_0'(k_0 r_s)}{j\omega\rho_1} u_a e^{j\omega t}$, respectively.

Thus, the ehancement of the measured pressure value by the high-refractive-index elements from the point A to B is given by:

$$\eta = \frac{|P_{\text{SD}}|}{|P_0|} = \frac{\rho_1 |\mathbf{T}_{G\alpha}|}{\rho_0 |\mathbf{T}||H_0(k_0 d_t)|} \tag{6}$$

The results at different distance d_t are shown in Fig. S1, which clearly confirm the efficient enhancement in both near field in close proximity much shorter than a wavelength and far field much longer than a wavelength.

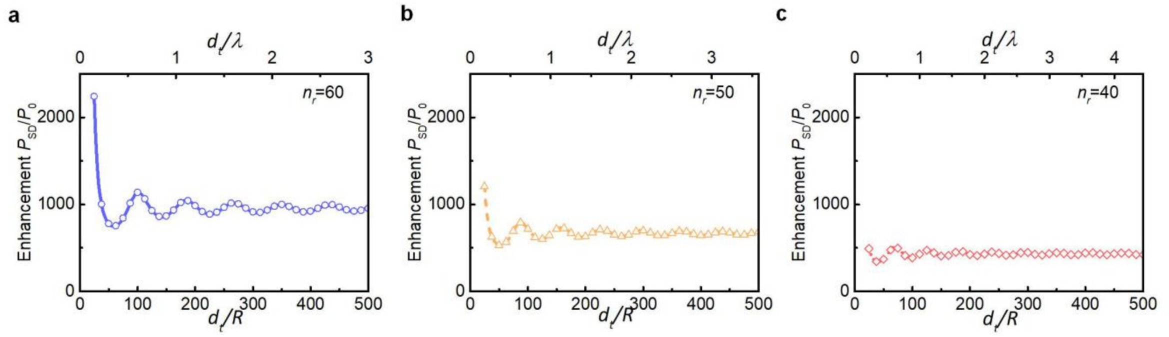

Figure S1: Enhancement for different relative refractive index n_r. Enhancement for **(a)** n_r = 60, **(b)** n_r = 50, and **(c)** n_r = 40 at different distances d_t in terms of both the element radius R (bottom x-axis) and operating wavelength λ (top x-axis).

Note 2. RWM at higher-order monopolar modes

In addition to the basic monopole acoustic mode, the system composed of high-refractive-index particles are capable of exciting higher order monopole modes. Figure S2(a) shows the theoretical and simulated enhancement spectra P_{SD}/P_0 when placing two particles ($n_r = 60$) at the launch and receiving ends. The spectrum exhibits the monopole resonance, the second monopole resonance and the third monopole resonance at 518 Hz, 1205 Hz and 1901 Hz, respectively. It is worth noting that the enhancement value is highest at the monopole resonance mode and decreases with the increasement of the order. The corresponding pressure field distributions of these three modes at the receiving end are shown as Fig. S2(b)-S2(d). In each order of monopole resonance mode, the sound energy is highly localized within the high-refractive-index particle and the first monopole resonance mode exhibits the strongest intensity of the sound field. Thus, we focus on the first-order monopole resonance in the manuscript due to its strongest capability to enhance the detected pressure value while manipulate sound waves with larger wavelength.

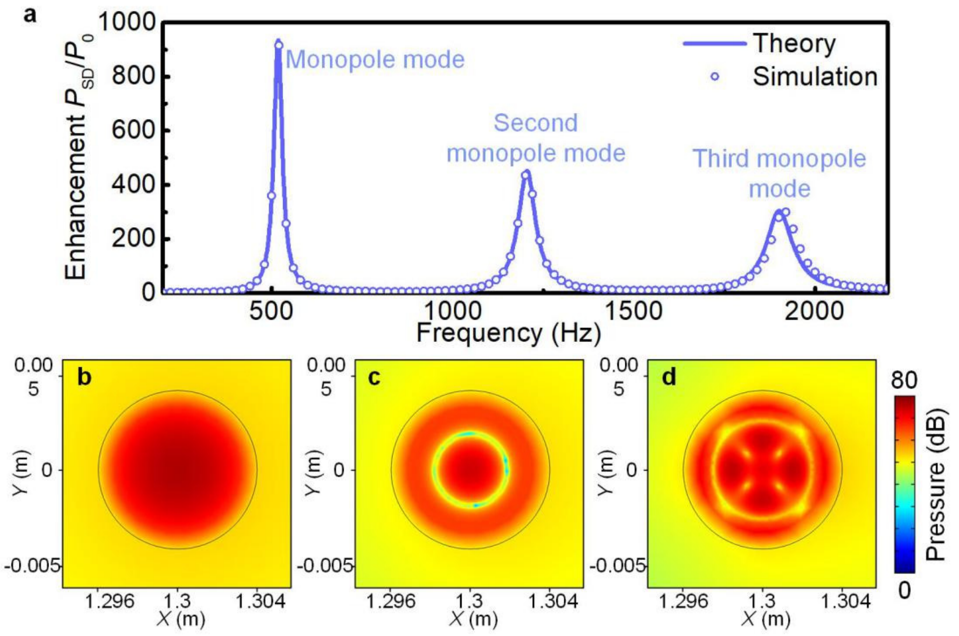

Figure S2: RWM at higher-order monopolar modes. **(a)** Theoretical and simulated enhancement spectra P_{SD}/P_0 when placing high refractive index particles ($n_r = 60$) at the launch and receiving ends. **(b)-(d)** Corresponding pressure field distributions of the first-order, second-order, and third-order monopolar mode.

Note 3. Influence on radiation impedance of the speaker

The radiation capability of a sound source is normally characterized in terms of the real part of the acoustic radiation impedance Z, i.e., the radiation resistances Re(Z). For a given source like a speaker, the acoustic radiation resistances calculated from the complex ratio of sound pressure on the surface of a source to the corresponding normal velocity of the source with and without the metacavity are shown in Fig. S3. The results clearly exhibit a significant enhancement at the resonance frequency in the presence of the metacavity in comparison to the bare source. The changes in the radiation impedance thus indicate that more energy can be emitted to far field.

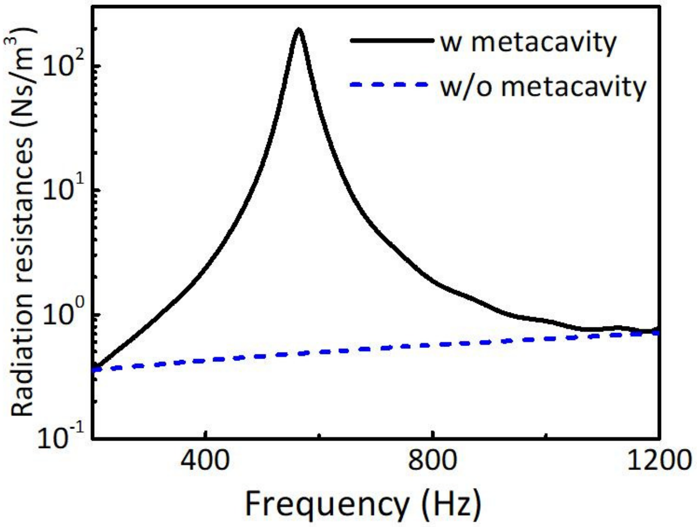

Figure S3: Sound source's radiation resistance Re(Z) with and without the metacavity.

Note 4. Experimental setup

For the experimental setup and measurement, we employed a balanced armature speaker (Knowles, Model ID DWFK-31785-000, 5 × 2.7 × 3.9 mm^3) as the monopole source, with the speaker mouth embedded and sealed into a hole (6 mm radius) at the center of the element **S** (see Fig. S4). The miniature dimensions of this speaker and its efficient response at required low-frequency range make it a legitimate point source for experimental measurements. A condensed microphone (Brüel & Kjær type-4939) was placed at the center of the element D to acquire the excited sound pressure signal, that is 1.3 m away from the speaker. To obtain the gain spectra, the pressure responses are measured by frequency sweeping from 200 to 1000 Hz with an interval of 5 Hz. The Mie-resonance based meta-cavities S and D are made of photosensitive resin and manufactured via 3D printing technology. Sound absorbing sponges are placed at the exterior boundaries of the waveguide to eliminate the effects of reflected waves.

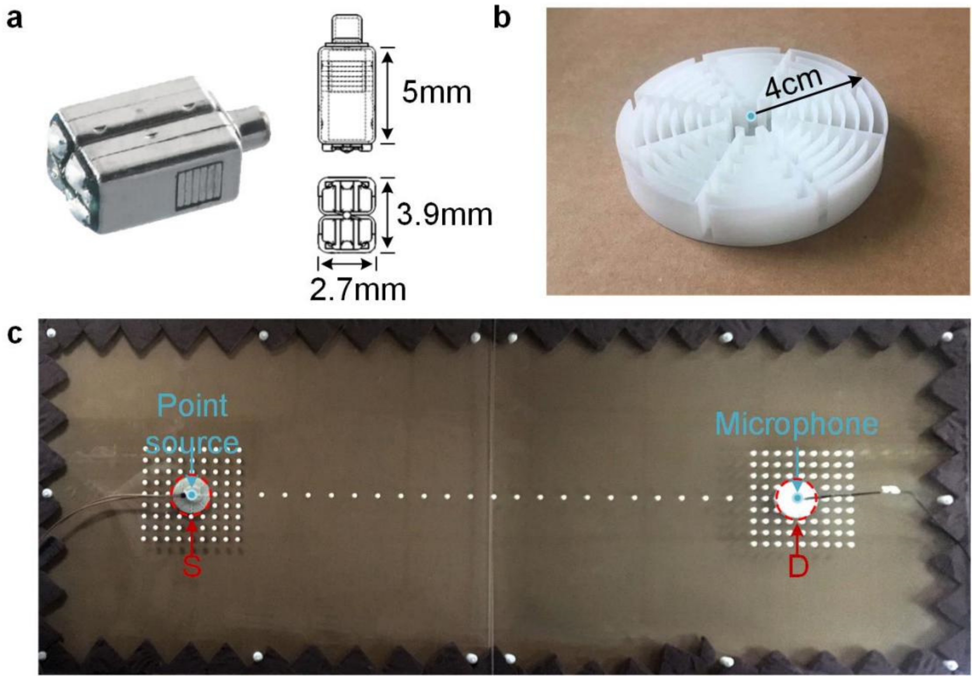

Figure S4: Experimental setup. (a) A balanced armature speaker used as the monopole source with a size of 5×2.7×3.9 mm^3. **(b)** Photograph of the Mie meta-cavity with a radius of 4 cm. **(c)** Top view of the experimental setup. The speaker mouth and the condenser microphone are embedded and sealed into the holes at the center of the meta-cavity S and D, respectively.

Note 5. Describing the physical model with a rigorous acoustic scattering theory

The meta-cavity labyrinth structure can be simplified into a previously demonstrated physical equivalent with an inner core of background medium, an outer layer (yellow region) of equivalent medium and a virtual layer (gray region), as shown in Fig. S5(a) [1]. The radius of the outer layer is $r_3 = r_2 + (R - r_2)\eta$ with η the filling ratio of the narrow channels. The virtual layer is introduced to leave the total radius of the element unchanged, which has the identical fields at the two interfaces. The transfer system can be divided into 5 different regions using the simplified model: the inner and outer layer of the emitting (receiving) region and the free space. Setting $\mathbf{r} = (x, y) = (r, \theta)$, the pressure field P obeys the 2D Helmholtz equation $(\nabla^2 + k^2)P = 0$, which can be decomposed into the incident (outgoing) and scattering cylindrical waves represented by Bessel(J_m) and Hankel(H_m) functions

$$P(r) = \begin{cases} \sum_m A_m H_m(k_0|\mathbf{r}_\mathrm{A}|) + B_m J_m(k_0|\mathbf{r}_\mathrm{A}|)\,, r_s < |\mathbf{r}_\mathrm{A}| < r_2 \\ \sum_m C_m H_m(k_1|\mathbf{r}_\mathrm{A}|) + D_m J_m(k_1|\mathbf{r}_\mathrm{A}|)\,, r_2 < |\mathbf{r}_\mathrm{A}| < r_3 \\ \sum_m E_m H_m(k_0|\mathbf{r}_\mathrm{A}|) + F_m J_m(k_0|\mathbf{r}_\mathrm{A}|) = \sum_m G_m J_m(k_0|\mathbf{r}_\mathrm{B}|) + K_m H_m(k_0|\mathbf{r}_\mathrm{B}|)\,, |\mathbf{r}_\mathrm{A}| > R \; and \; |\mathbf{r}_\mathrm{B}| > R \\ \sum_m L_m J_m(k_1|\mathbf{r}_\mathrm{B}|) + M_m H_m(k_1|\mathbf{r}_\mathrm{B}|)\,, r_2 < |\mathbf{r}_\mathrm{B}| < r_3 \\ \sum_m N_m J_m(k_0|\mathbf{r}_\mathrm{B}|), |\mathbf{r}_\mathrm{B}| < R \end{cases}$$

A_m~N_m are the mth-order expansion coefficients for each layer. The pressure value at the receiving position $|\mathbf{r}_\mathrm{B}| = 0$ is $P_\mathrm{SD} = \sum_m N_m J_m(0) = N_0$, which is the 0th-order expansion coefficient of the incident waves in meta-cavity D. The value should be highest in the case of monopole resonance.

In the outer space, the incident wave of the meta-cavity D should be an outgoing wave from the meta-cavity S. Correspondingly, the incident wave of the meta-cavity S should be a scattered wave from the meta-cavity D. It can be expressed as:

$$\begin{cases} G_0 \approx E_0 H_0(k_0|\mathbf{d}|) \\ F_0 \approx K_0 H_0(k_0|\mathbf{d}|) \end{cases}$$

Combining the continuities of $P(r)$ and $1/\rho \cdot \partial \mathrm{P}/\partial \mathrm{r}$ at the interfaces $|\mathbf{r}_\mathrm{A}| = r_2$, $|\mathbf{r}_\mathrm{A}| = R$, $|\mathbf{r}_\mathrm{B}| = r_2$ and $|\mathbf{r}_\mathrm{B}| = R$, the 0th-order coefficients are obtained after solving:

$$\mathbf{T} \times [B_0 \quad C_0 \quad D_0 \quad E_0 \quad F_0 \quad G_0 \quad K_0 \quad L_0 \quad M_0 \quad N_0]^T = A_0 \boldsymbol{\alpha}$$

where $\boldsymbol{\alpha} = \left[H_0(k_1 r_2) \quad \frac{k_0}{\rho_0} H_0'(k_1 r_2) \quad 0 \quad 0 \quad 0 \quad 0 \quad 0 \quad 0 \quad 0 \quad 0\right]^T$ and the matrix $\mathbf{T}$ is expressed as:

$$T = \begin{bmatrix} -J_0(k_0r_2) & H_0(k_1r_2) & J_0(k_1r_2) & 0 & 0 & 0 & 0 & 0 & 0 & 0 \\ -\frac{k_0}{\rho_0}J_0'(k_0r_2) & \frac{k_1}{\rho_1}H_0'(k_1r_2) & \frac{k_1}{\rho_1}J_0'(k_1r_2) & 0 & 0 & 0 & 0 & 0 & 0 & 0 \\ 0 & H_0(k_1r_3) & J_0(k_1r_3) & H_0(k_0R) & J_0(k_0R) & 0 & 0 & 0 & 0 & 0 \\ 0 & \frac{k_1}{\rho_1}H_0'(k_1r_3) & \frac{k_1}{\rho_1}J_0'(k_1r_3) & \frac{k_0}{\rho_0}H_0'(k_0R) & \frac{k_0}{\rho_0}J_0'(k_0R) & 0 & 0 & 0 & 0 & 0 \\ 0 & 0 & 0 & 0 & 0 & J_0(k_0R) & H_0(k_0R) & J_0(k_1r_3) & H_0(k_1r_3) & 0 \\ 0 & 0 & 0 & 0 & 0 & \frac{k_0}{\rho_0}J_0'(k_0R) & \frac{k_0}{\rho_0}H_0'(k_0R) & \frac{k_1}{\rho_1}J_0'(k_1r_3) & \frac{k_1}{\rho_1}H_0'(k_1r_3) & 0 \\ 0 & 0 & 0 & 0 & 0 & 0 & 0 & J_0(k_1r_2) & H_0(k_1r_2) & -J_0(k_0r_2) \\ 0 & 0 & 0 & 0 & 0 & 0 & 0 & \frac{k_1}{\rho_1}J_0'(k_1r_2) & \frac{k_1}{\rho_1}H_0'(k_1r_2) & -\frac{k_0}{\rho_0}J_0'(k_0r_2) \\ 0 & 0 & 0 & H_0(k_0d_t) & 0 & -1 & 0 & 0 & 0 & 0 \\ 0 & 0 & 0 & 0 & -1 & 0 & H_0(k_0d_t) & 0 & 0 & 0 \end{bmatrix}$$

According to the above equation, we can obtain the detected pressure as: $P_{\mathrm{SD}} = N_0 = |\mathbf{T}_{N\alpha}|/|\mathbf{T}|A_0$. The ehancement of the measured pressure value is:

$$\eta = \frac{|P_{\mathrm{SD}}|}{|P_0|} = \frac{|\mathbf{T}_{N\alpha}|}{|\mathbf{T}||H_0(k_0 d)|}$$

Figure S5(b) shows the theoretical transfer enhancement P_{SD}/P_0, which is consistent with the results in Fig. 3(e) in the manuscript. The minor difference between the peak and the resonant frequency may be attributed to the parameter approximation of the simplified equivalent model. We have also studied the enhancement value at different distances d_t and the results are given in Fig. S5(c). This value fluctuates when d_t is small and tends to stabilize as d_t increases.

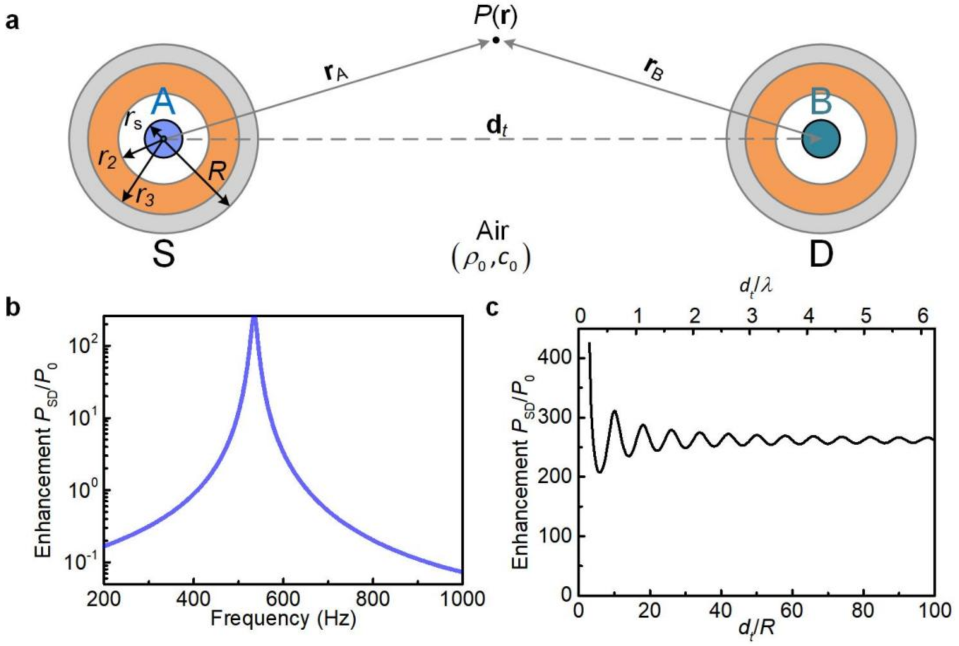

Figure S5: Theoretical model for the physical RWM system. (a) Schematic of the system setup. A is a single point-like sound source of radius r_s, which outputs a sine harmonic wave. B is a condensed microphone which picks up the sensing signal. S and D are respectively the equivalent model of the source and detector meta-atoms referred to in the text, aligned with parallel axis. **(b)** Theoretical transfer enhancement from A to B with both S and D compared with the case without S and D. Here the transfer distance $d_t = 32.5R$. **(c)** The value of enhancement at different distances d_t in terms of both the element radius R (bottom x-axis) and operating wavelength λ (top x-axis).

Additionally, a careful analysis of the two efficiencies P_{S}/P_0 and P_{D}/P_0 as to how they relate to the overall performance P_{SD}/P_0 is presented. We compute the enhancement factor P_{S}/P_0 for the enclosed speaker with the bare microphone and equivalently P_{D}/P_0 for the opposite configuration, finally to be able to compare them to the system where both are enclosed with the respective enhancement factor P_{SD}/P_0. In doing so, we varied the separation d_t/λ between microphone and loudspeaker and defined the factor $P_{\mathrm{SD}}P_0/P_{\mathrm{S}}P_{\mathrm{D}}$ to be able to assess whether the process is a product of emission and reception.

In the near-field regime, Fig. S6 clearly displays how the enhancement when both elements are enclosed by the RWM exceeds the product of the individual processes since $P_{\mathrm{SD}}P_0 > P_{\mathrm{S}}P_{\mathrm{D}}$, which stems from the strong monopole-monopole interaction occurring between two such resonators that is giving rise to the pronounced sound field enhancement including pressure oscillations. On the other hand, in the far-field the system can be broken down into two cascaded processes of emission and receiving the signal, and the total transfer efficiency is approximately the product of two efficiencies, $P_{\mathrm{SD}}P_0 \approx P_{\mathrm{S}}P_{\mathrm{D}}$.

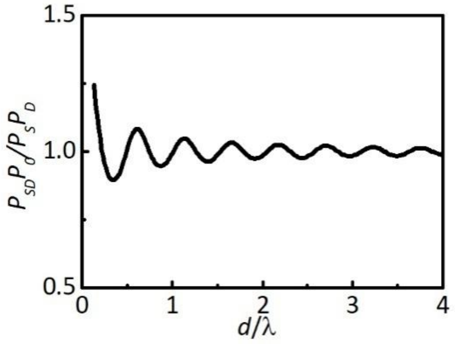

Figure S6: Contrast factor $P_{\mathrm{SD}}P_0/P_{\mathrm{S}}P_{\mathrm{D}}$ between the enhancement when both elements are enclosed by the RWM and the product of the individual processes.

Note 6. Comparison with the classic acoustic Helmholtz resonators

To shed more light on the superiority of the Mie meta-cavities over classic acoustic elements, Fig. S7 shows the enhancement spectra P_{SD}/P_0 when replacing the Mie meta-cavities with uniform-sized Helmholtz resonators in ideal lossless model and actual lossy model. The radius of the Helmholtz resonator is $R = 4\mathrm{cm}$ and the propagation spacing is set as $d_t = 32.5R$. We investigated various relative angle between the two slits of the Helmholtz resonators, and depicted here the results of face-to-face (red solid line) and back-to-back (blue dashed line) configurations for illustration. The enhancement spectrum P_{SD}/P_0 when employing the Mie meta-cavities is also depicted as the yellow dotted line for comparison.

The following remarks should be noted: (1) The Helmholtz resonator pair only reaches 81.6 in enhancement, which is far less than the performance of the Mie meta-cavities [see Fig. S7(a)]. The enhancement can be further increased by tailoring a larger refraction index. Taking the absorption into consideration [see Fig. S7(b)], the proposed Mie meta-cavities still displays a significantly higher enhancement compared to Helmholtz resonators. The maximum enhancement with Mie meta-cavities including loss is 157 (60.6 % of the value 259 without loss), which is far greater than 31.7 (38.8% of the value 81.6 without loss) with Helmholtz resonators. (2) In the case of Helmholtz resonator, the element configuration has a great influence on the enhancement performance, as the maximum amplification reduces from 81.6 in face-to-face configuration to 38.5 in back-to-back configuration. In contrast, the omnidirectional performance in the condition of Mie meta-cavities is almost independent of element configuration, owing to the high symmetry of the meta-cavities. (3) The Mie system hosts several monopolar and multipolar resonances in comparison the Helmholtz resonator with one monopolar resonance only, which make the use of the RWM much more flexible (see Supplementary Note 2).

In principle, the metacavity and Helmholtz resonator are two distinct structures with localized resonances. Contrary to the inertial resonance nature of the Helmholtz resonator that creates a localized monopole resonance in its neck-cavity, which acts as mass-spring oscillator, the metacavity achieves a strong monopolar response via intrinsic resonances originating from the Mie scattering of the high-index geometry ($n_r > 1$). Further, the fluid particles at the Helmholtz resonator's neck yields free-space sound radiation similar to an open-ended pipe in contrast to the metacavity, whose coherent symmetrical radial oscillation stems from a monopolar Mie resonance when the effective wavelength equals the resonator diameter. Those fundamentally different resonance-origins when comparing the two devices, explain the distinct performances when it comes to the acoustic pressure enhancement.

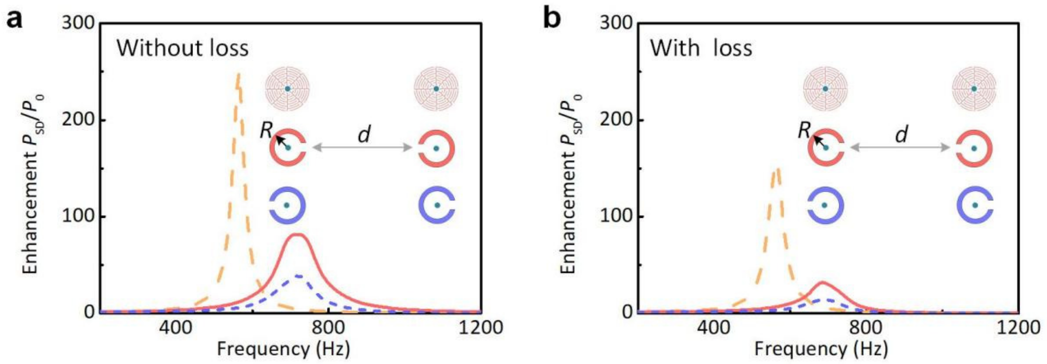

Figure S7: Comparison with the classic Helmholtz resonators with and without loss. (a) Enhancement spectrum P_{SD}/P_0 in the case without thermal-viscous loss when placing the Helmholtz resonators pairs both face-to-face (red solid line) and back-to-back (blue short dashed line) in comparison to the RWM (yellow dotted line). Inset: schematic diagram of the configurations. **(b)** Same as **(a)** but for results including loss.

Note 7. Sound pressure field emitted from a weak sound source in free space

Figure S8 gives the sound pressure fields emitted from a weak sound source in the condition without and with the RWM system. The volume flow rate per unit length out from the source is $Q_2 = Q_1/157$. The detected pressure is only 16.1 dB for the free space case [see Fig. S8(a)], which is too weak to detect even in an extremely quiet environment, let alone in ordinary noisy background with low SNR. For comparison, the RWM system can enhance the detected sound pressure emitted from the same weak sound source up to 60 dB [see Fig. S8(b)]. Note that the enhancement in the receiving location is much larger than that in exterior area. As a result, we can achieve efficient signal amplification for the specified location while keeping the other areas relatively quiet, no matter using the same weak sound source.

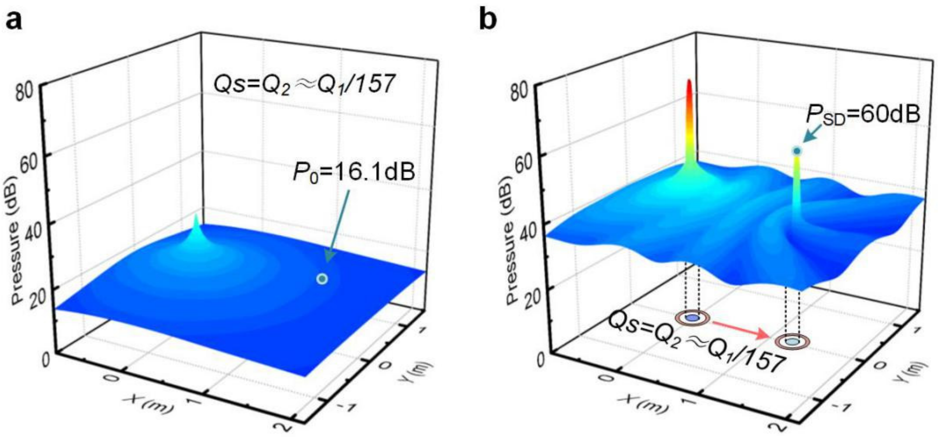

Figure S8: Comparison to a weak sound source with the volume flow rate $Q_2 = Q_1/157$. **(a)** Sound pressure field emitted from the weak sound source directly, in free space without RWM system. The detected pressure at the receiving point is 16.1 dB. **(b)** Sound pressure field of the same weak sound source using RWM system. The detected pressure reaches up to 60 dB.

Note 8. System robustness

System robustness is highly desirable for the practical realization of relevant functional devices. We have studied the performance of the RWM system in the condition of large obstacle or random scatterers. The results show good robustness against various variations and objects.

Figure S9(a) shows the enhancement spectrum P_{SD}/P_0 when placing a large solid obstacle in the propagation path between the launch and receiving ends to block the pathway of direct sound. The obstacle is placed 15 cm away from the receive ends and has a length of 0.6 m and a width of 0.1 m. For the case without the obstacle, the enhancement value can reach up to 259 [see Fig. 3(e)] and the value for the case with the obstacle is 254. The maximum enhancement value is almost the same in both cases. For comparison, the pressure field distributions at the frequency of 563 Hz for the obstacle case with and without the RWM are shown in Fig. S9(b) and S9(c). Without the RWM device, the sound intensity is extremely low at the receiving ends as the obstacle blocks the transmission of most sound waves. On contrary, for the result with RWM system, the emission efficiency is improved by the meta-cavity at the launch end and the sound energy is strongly localized into the receiving area by the meta-cavity at the receiving end.

We also examine the robustness against the scattering layer and the results are shown in Fig. S9(d)-S9(e). For illustration, a random scattering layer consists of 30 randomly distributed rigid scatterers with sound hard boundary and a radius of 4 cm is placed in the middle of the launch and receiving ends. The scattering layer has a total length of 0.6 m and a total width of 1 m, which makes sound wave propagation more complicated. However, the RWM system still show an excellent performance in enhancing the intensity at the receiving end [see Fig. S9(d)]. The maximum enhancement reaches 293, which is even higher than the free space case. The sound pressure field distributions shown in Figs. S9(e) and S9(f) intuitively confirm the distinct sound pressure fields especially at the receiving end.

For comparison, Figure S10 shows the enhancement spectrum P_{SD}/P_0 of the system consisting of Helmholtz resonators in the condition with and without the large size solid obstacle. The obstacle has a significant negative influence on the performance of the system as the maximum amplification reduces by 66%. Thus, the proposed Mie meta-cavities also exhibit the superiority of better robustness compared with classic acoustic Helmholtz resonators.

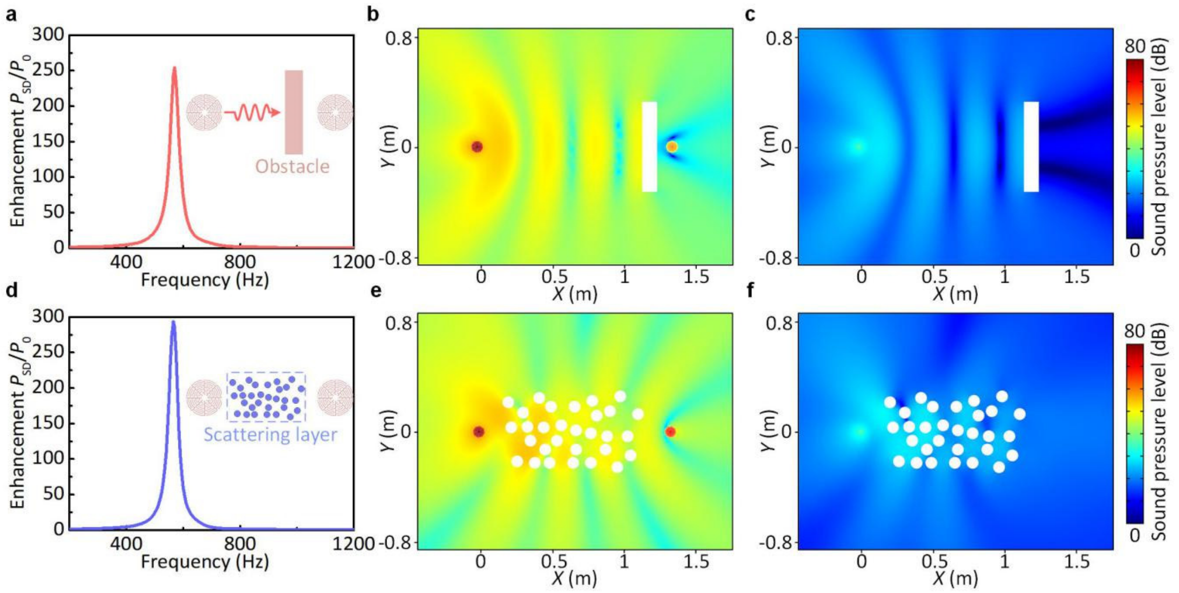

Figure S9: Robustness of the RWM system. (a) Enhancement spectrum P_{SD}/P_0 when placing a large size solid obstacle in the propagation path between the launch and receiving ends. Corresponding pressure field distributions at 563 Hz for the case **(b)** with and **(c)** without the RWM system for the obstacle case. **(d)-(f)** Same as **(a)-(c)** but for a random scattering layer in the propagation path between the launch and receiving ends.

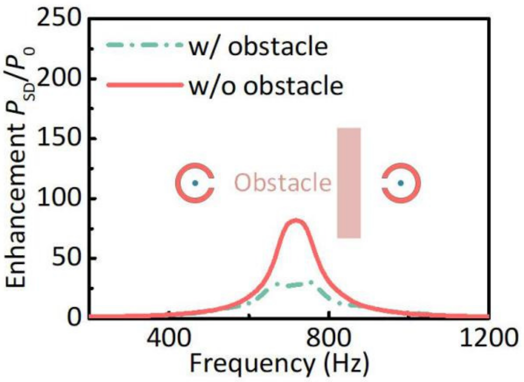

Figure S10: Comparison with the system consisting of classic Helmholtz resonators. Enhancement spectrum P_{SD}/P_0 in the condition with and without a large size solid obstacle.

Note 9. Multi-target remote whispering

As shown in Fig. S11(a), receiving regions I, II, and III and the point source in the center are covered by proposed Mie resonance meta-cavities. The volume flow rate per unit length out from the source is $Q_2 = Q_1/157$ and the distances are $d_1 = 1.3$ m, $d_2 = 0.5$ m and $d_3 = 3$ m for illustration. Figure S11(b) gives the sound pressure field radiated from the weak sound source using the multi-target remote whispering. The detected sound pressure level in the center of regions I, II, and III are 60.6 dB, 63.2 dB and 57.7 dB, which is significantly higher than the average value of 38.3 dB in surrounding environment. Thus, we can amplify signals in several designated locations while keeping the other space quiet in real time using the proposed scheme.

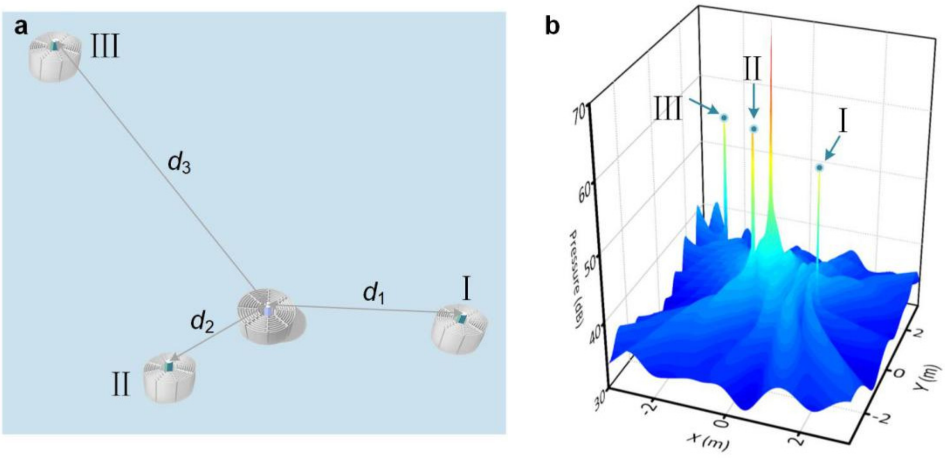

Figure S11: Multi-target remote whispering. (a) Schematics of the system setup. Receiving regions I, II, and III and the point source in the center are enclosed by Mie meta-cavities. The distances are set as $d_1 = 1.3$ m, $d_2 = 0.5$ m and $d_3 = 3$ m for illustration. The volume flow rate per unit length out from the source is $Q_2 = Q_1/157$. **(b)** Sound pressure fields.

Note 10. Overcoming the conventional acoustic detection limit

The volume of the noise speaker in Fig 5(a) was adjusted to control the signal-to-nose ratio (SNR) of the detected signal. Figure S12 gives the comparison of the SNR of the signal measured in free space and in the RWM system. The blue solid line is the fitted curve with a slope of about 116, indicating more than 116 times enhancement (that is, about 20.7 dB) of SNR achieved in the RWM system under different background noise conditions, compared with that obtained in free space. This will enable the RWM system to detect weak acoustic signals below the detection limit of a conventional acoustic sensor (that is, SNR<1 in free space).

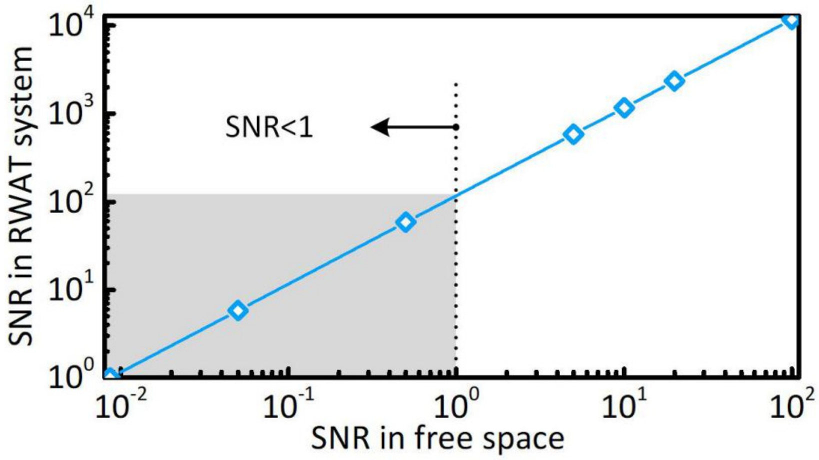

Figure S12: SNR measured in the RWM system compared with that obtained in free space. The input signal from the speaker was a series of Gaussian pulses with a center frequency of 563 Hz and a band width of 75 Hz. In the gray highlighted zone, the simulated free-space SNR is < 1, indicating that the input signal is below the detection limit of conventional acoustic detection system.

Note 11. Anti-interference remote whispering in an extreme case

As shown in Fig. S13(a), we also investigate the performance of the proposed RWM system in an extreme case when the strong external interference source is placed between the sound source and the receiving location. The distance between the noise and the receiving location is set as 0.325m, which is only a quarter of the distance between the sound source and the location. Figures S13(b) and S13(c) show the time domain and frequency domain pulse signal measurements in free space (top) and in by using the RWM (bottom). Compared with the results in Fig. 5(b) and 5(c), the influence of the noise is stronger in free space while the detected signal in RWM system does not suffer from any obvious distortions.

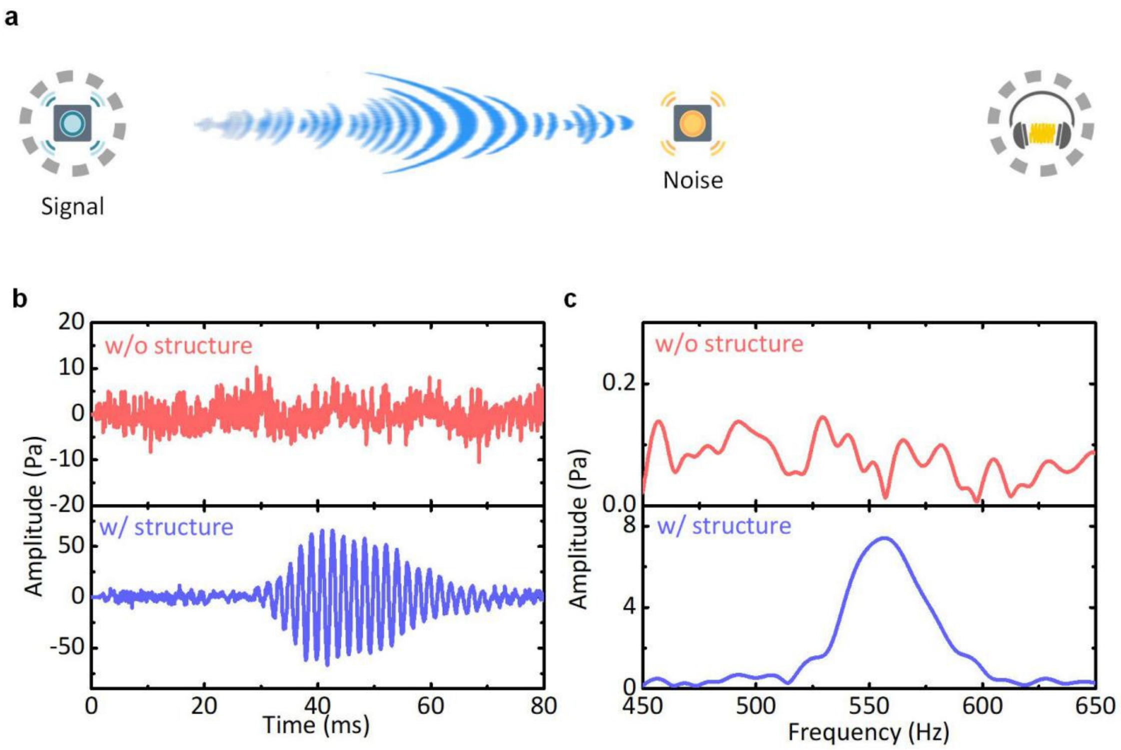

Figure S13: Anti-interference remote whispering in an extreme case. (a) A weak sound source emits a series of Gaussian modulated acoustic pulses with center frequency at 563 Hz and bandwidth of 75 Hz (blue). A strong external interference source radiates broadband white noise (yellow) is placed between the sound source and the receiving location to mask the desired weak signal. **(b)** Pressure waveforms in the time-domain and **(c)** corresponding FFT spectra measured in free space without the RWM (upper panel) and with the RWM (lower panel) structure.

Note 12. High transfer efficiency for sound signal & information

The proposed RWM device can exhibit valuable high efficiency from the viewpoint of signal & information transfer and detection. Concerning such matter of efficiency, please note that the SNR obtained by RWM could be significantly higher than that obtained by a bare receiver of the same surface area.

To corroborate this claim, we have investigated the SNR by taking the receiver area into account. As shown in the schematic diagrams in Figs. S14(a) and S14(b), we calculated the SNR of the sound signals received by a standalone small receiver with a diameter of 0.64 cm (the same size as the 1/4-inch microphone used in experiments) and a standalone fictitious large receiver with a diameter of 8 cm (the same size as the Mie resonator), respectively, all in the absence of the metamaterial. Here, a series of Gaussian modulated sinusoidal pulses (blue) is generated from the source speaker to mimic a whispered weak sound signal, which is mixed with broadband white noise (yellow) radiated from a strong external interference source. The middle and bottom row in Fig. S14 show the corresponding calculated time domain and frequency domain signals. It can be seen that the size of the receiver has no obvious effect on the quality of the receiver signal, in spite of the obvious amplitude differences that however carry no additional information. Increasing the size of the receiver from 0.64 cm to 8 cm only achieves a minor SNR improvement of about 0.035 dB, which is negligible in practice. For comparison, the unique enhancement effect can be achieved by the small receiver enclosed by our RWM system subject to the same source signal and noise, as shown in Fig. S14(c). Note that the RWM brings an SNR improvement of about 20.7 dB, which signifies a remarkable increase. This SNR improvement originates from the enhanced directionality and frequency selectiveness induced by the monopole-monopole interaction of two Mie-resonators, which is highly efficient and NOT supported by a receiver of arbitrary surface area.

Additionally, to further elaborate on the high efficiency of SNR enhancement by our RWM compared to the bare receiver, Fig. S15 presents the distinction between the SNR of the signal obtained by our RWM system with a small receiver and the SNR obtained in free space by a large receiver. The blue solid line is the fitted curve with a slope of about 116, which indicates a 116 times enhancement (about 20.7 dB) of the RWM SNR under different background noise conditions, compared with the SNR of the same small but bare receiver. This enhancement enables the RWM system to detect extremely weak acoustic signals far below the detection limit of a conventional acoustic receiver (that is, SNR < 1 in free space), which exhibit high efficiency in signal & information transfer and detection. On the contrary, the purple solid line has a slope of only 1.008, indicating almost the same SNR obtained by both the large area receiver and small area receiver without using the metamaterial.

In conclusion, we have clearly demonstrated a unique and substantial improvement of the SNR which is not achievable by an arbitrary regular receiver, confirming that the practical prospects for remote whispering sound transfer are extremely efficient and realistic beyond its physics related beauty.

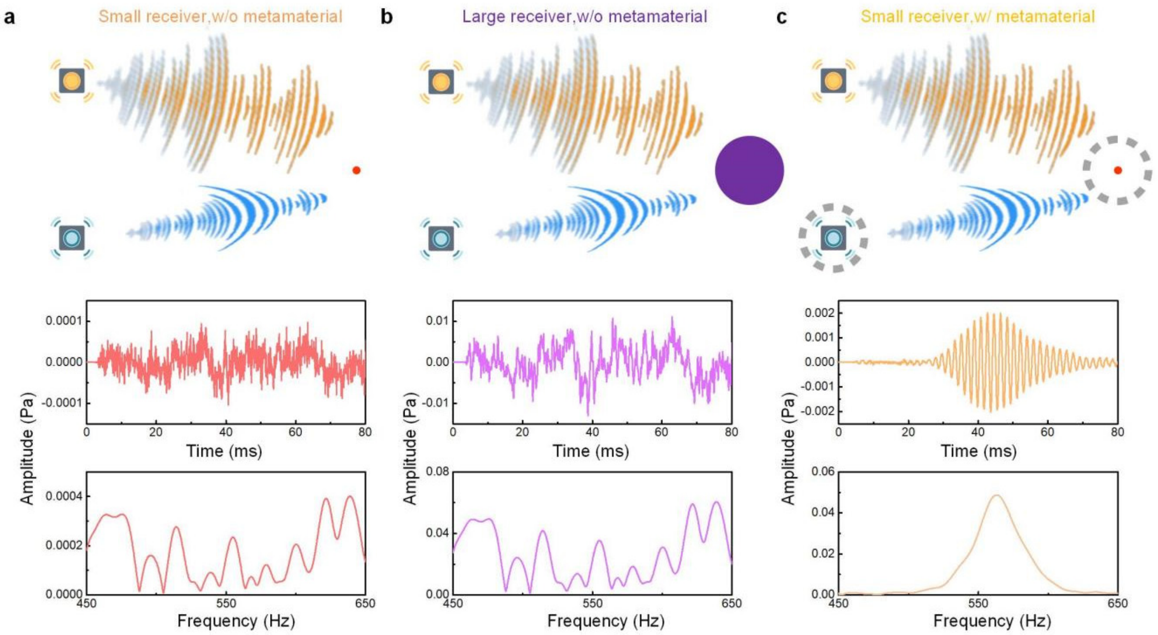

Figure S14: Neither (a) a small receiver with a diameter of 0.64 cm (the same size as the microphone) nor (b) a large receiver with a diameter of 8 cm (the same size as the Mie resonator) can pick up weak signals that interfere with strong white noise in free space without using a metamaterial. (c) With the help of RWM system, the small receiver can pick up efficiently weak signals in the presence of strong white noise. Top row: schematic diagrams. Middle row: time-domain pressure waveforms of the received signals. Bottom row: corresponding FFT spectra.

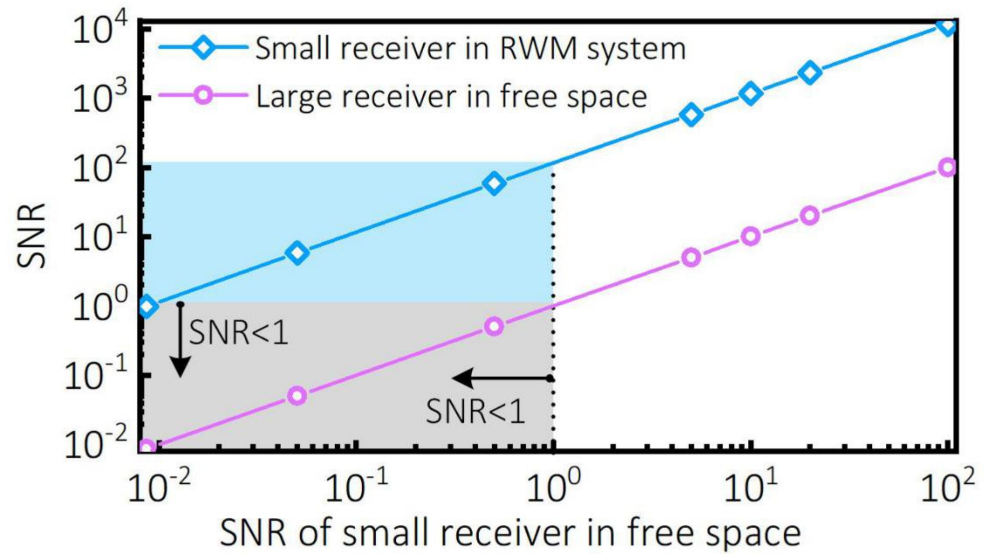

Figure S15: Computed SNRs for the RWM with small receivers and in free space with large receivers. Both predictions are plotted against the SNR in free space with small receivers. The grey shaded area marks the loss dominated region where the SNR < 1 of the large receiver in free space. The blue area indicates the SNR enhancement, i.e., SNR > 1, of the RWM in comparison to free space. Here, the SNR of the RWM system has improved by over 20 dB as compared to a large receiver without using a metamaterial. This representation indicates a superior detection of weak sound signals within the limiting bounds of conventional acoustic detection systems, confirming the remarkable high transfer efficiency of the RWM for sound signal & information.

Note 13. System reconfigurability

System reconfigurability is highly desirable for the practical realization of relevant functional devices. Certain limited reconfigurations of the device can be provided even if the physical structure of the RWM remains unchanged.

For illustration, the working frequency of the RWM can be simply reconfigured by external temperature variations, due to the effect that both the speed of sound in air c_0 and mass density ρ_0 change with temperature. The value of c_0 and ρ_0 can be computed from the measured temperature according to the following expressions:

$$c_0 = 20.047\sqrt{273.15 + T} \ ,$$

$$\rho_0 = 1.290\left(\frac{P}{101.325}\right)\left(\frac{273.15}{273.15+T}\right),$$

where P = atmospheric pressure in unit of kPa and T = ambient temperature in unit of ℃. Thus, the characteristic impedance of air, $\rho_0 c_0$, decreases with an increment of T.

To elaborate on the achievable reconfigurability of RWM, Fig. S16 shows the adjustable range of the operation frequency, which can be tuned from 534 Hz to 574 Hz when T changes from -10 ℃ to 30 ℃. Note that even a wider adjustable range can be obtained by further changing the value of T and/or P.

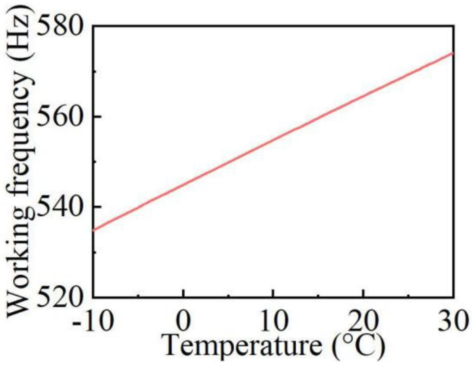

Figure S16: Limited reconfigurability of the RWM working frequency under an applied temperature variation.

Note 14. RWM system for underwater sound

Full-wave numerical simulations are conducted to characterize the acoustic response of a RWM system for underwater sound. As shown in Fig. S17(a), the external surface of each meta-cavity is covered with a rubber coating layer to isolate the air medium inside the structure from exterior water background (in order to avoid the serious viscous effect and thin-wall vibration when the meta-cavity is filled with water instead, while significantly lower the working frequency so as to make the meta-cavity in deep subwavelength scale). The simulation is performed by the acoustic-solid interaction mode in COMSOL to characterize the intensive interaction between fluid and solid. For illustration, the transfer distance is set to 100 m, while the inner and outer radii of the rubber layer are only 4 cm and 4.5 cm, respectively. The Young's modulus and Poisson's ratio of the rubber layer are set as 0.6 MPa and 0.49, respectively. Figure S17(b) presents the simulated transfer enhancement P_{SD}/P_0 of the proposed underwater RWM system. The spectra exhibit a maximum enhancement peak of 12.97 at the extremely low frequency of 26.5 Hz, and the corresponding sound pressure field is shown in Fig. S17(c). The enhancement of the detected signals and the reduction of the ambient sound leakage can also be observed.

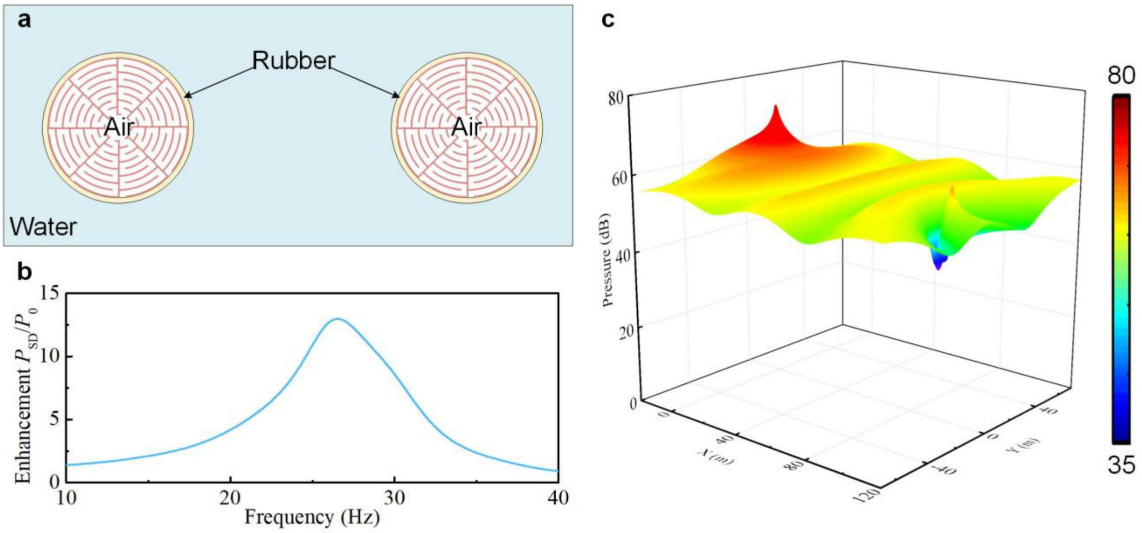

Figure S17: (a) Schematic of an underwater RWM system. (b) Transfer enhancement P_{SD}/P_0 of the underwater system. (c) Sound pressure field of the underwater RWM system at the frequency of 26.5 Hz.

Note 15. RWM system for electromagnetic signal

Moreover, our presented approach can in fact be extended beyond the sole use for acoustic wave transfer. Under the same principle, simulations of the RWM but for electromagnetic (EM) signals are shown in Fig. S18, which depicts how the received EM signal can be substantially enhanced with co-existing dipolar or quadrupolar modes in RWM system (blue solid curve) than ordinary detectable signals in free space (red dashed curve).

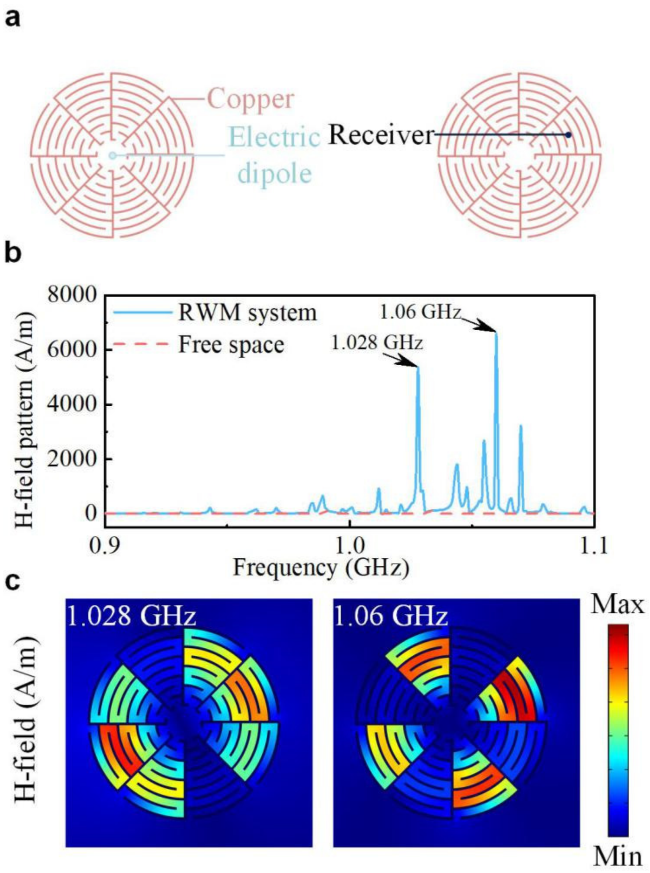

Figure S18: (a) Schematic of RWM system for electromagnetic signals, in which the meta-cavities can be fabricated using metals such as copper. (b) Comparison between received signal with RWM system and in free space. (c) H-field of the RWM system at the frequency of 1.028 GHz and 1.06 GHz.

Supplementary References

[1] Y. Cheng, C. Zhou, B. G. Yuan, D. J. Wu, Q. Wei, and X. J. Liu, Nat. Mater. 14, 1013 (2015).
